# Common Complications and Cardiopulmonary Resuscitation in Patients with Left Ventricular Assist Devices: A Narrative Review

**DOI:** 10.3390/medicina59111981

**Published:** 2023-11-10

**Authors:** Jerica Zaloznik Djordjevic, Timur Özkan, Eva Göncz, Jus Ksela, Martin Möckel, Matej Strnad

**Affiliations:** 1 Department of Emergency Medicine, University Medical Centre Maribor, 2000 Maribor, Slovenia; 2 Department of Emergency and Acute Medicine, Charité—Universitätsmedizin Berlin, 10117 Berlin, Germany; 3 Department of Cardiovascular Surgery, University Medical Centre Ljubljana, 1000 Ljubljana, Slovenia

**Keywords:** cardiac arrest, cardiopulmonary resuscitation, ventricular assist device, heart failure, emergency medicine

## Abstract

Heart failure remains a major global burden regarding patients’ morbidity and mortality and health system organization, logistics, and costs. Despite continual advances in pharmacological and resynchronization device therapy, it is currently well accepted that heart transplantation and mechanical circulatory support represent a cornerstone in the management of advanced forms of this disease, with the latter becoming an increasingly accepted treatment modality due to the ongoing shortage of available donor hearts in an ever-increasing pool of patients. Mechanical circulatory support strategies have seen tremendous advances in recent years, especially in terms of pump technology improvements, indication for use, surgical techniques for device implantation, exchange and explantation, and postoperative patient management, but not in the field of treatment of critically ill patients and those undergoing cardiac arrest. This contemporary review aims to summarize the collected knowledge of this topic with an emphasis on complications in patients with left ventricular assist devices, their treatment, and establishing a clear-cut algorithm and the latest recommendations regarding out-of-hospital or emergency department management of cardiac arrest in this patient population.

## 1. Introduction

Heart failure (HF) remains a major medical burden, reaching epidemic proportions by affecting up to 50% of the general population with increasing incidence in individuals over 65 years of age [1,2]. Although progress in pharmacological and resynchronization therapy has led to reverse myocardial remodeling with symptomatic and survival benefits in this patient population, it is currently well accepted that heart transplantation and long-term mechanical circulatory support (MCS) represent a cornerstone in the management of advanced forms of the disease. The latter has become an increasingly accepted treatment modality due to the ongoing shortage of available donor hearts in an ever-increasing pool of patients [3]. Consequently, the extensive body of literature clearly indicates that MCS has been one of the most dynamic therapies in medicine in the last two decades, culminating in numerous advances in device technology and important observations made in selection strategies, indications, and management of patients undergoing implantation of durable MCS systems [4,5]. Yet, one area of post-implantation management remains scarcely addressed—managing cardiac arrest in these patients. This contemporary review aims to summarize collected knowledge and establish a clear-cut algorithm for the appropriate out-of-hospital or emergency department management.

## 2. Advanced Heart Failure

Traditionally, HF has been divided into distinct phenotypes based on the measurement of left ventricular ejection fraction (LVEF), thus compelling three major groups, namely HF with reduced EF (<40%), mildly reduced EF (41–49%), and preserved EF (>50%) [6]. Regardless of their LVEF, many patients with HF progress into a phase of advanced HF, which is a complex and heterogeneous clinical syndrome that develops due to progressive deterioration in the left ventricular function associated with LV remodeling and characterized by persistent symptoms despite maximal therapy [7,8] (Table 1). It is increasing in prevalence by about 1% annually, predominantly owing to the increasing age, demographics, and increasing prevalence of obesity in Western countries [1]. Worldwide, mortality among affected individuals exceeds 20% within 1 year after the first hospital or outpatient admission, and the majority of patients die within 5 years from the diagnosis [1,9,10]. A severely reduced LVEF is common but not required to diagnose advanced HF, as it may also develop in patients with diastolic dysfunction. 

The most common causes include ischemic heart disease, dilated cardiomyopathy, arterial hypertension, or end-stage valvular degeneration. There are several factors involved in cardiac remodeling, such as cell death (increased apoptosis and necrosis; decreased autophagy), oxidative stress (increased nicotinamide adenine dinucleotide phosphate (NADPH) oxidase; decreased antioxidative enzymes), inflammation, fibroblast proliferation, decreased contractility (decreased myosin and troponin phosphorylation) and neurohormonal activation (increased renin–angiotensin–aldosterone axis activation), which may all potentially lead to ventricular dysfunction [9]. As ventricular function deteriorates, the LV dilates and changes from an elliptical to a spherical shape. This increases wall stress, which then increases oxygen consumption, causes pathologic cardiomyocyte hypertrophy that further compromises contractile function, and can even induce functional mitral insufficiency. These changes lead to intractable HF. In addition, ventricular remodeling increases the tendency to develop ventricular arrhythmias [1,6,7,8,9,10,11].

## 3. Classifications

The New York Heart Association (NYHA) classification assesses the patient’s functional capacity and symptoms, progressing from no limitations of physical activity (Class I) to more significant limitations with less and less activity and eventually at rest (Class IV) (Table 2) [12]. 

Taking into account that NYHA Class IV represents a very heterogeneous group of patients, a more specific distinction among advanced HF phenotypes was proposed recently with the Interagency Registry for Mechanically Assisted Circulatory Support (INTERMACS) database, which was established in 2008 to facilitate the growth of MCS and improve patient outcomes. Paired with the registry’s first report was a proposal for a new system of classifying patients with advanced HF. Seven clinical profiles were designed to establish an enhanced method for characterizing patients requiring device therapy (Table 3). These profiles represent a stratification of symptomatic patients diagnosed with advanced HF despite optimal therapy. By documenting the profiles of patients undergoing device implantation, INTERMACS provides an opportunity to evaluate data and trends concerning surgical risks and indications [13]. 

## 4. Therapeutical Interventions

The backbone of pharmacological therapy for chronic HF remains a combination of beta-blockers, angiotensin-converting enzyme (ACE) inhibitors or angiotensin receptor blockers (ARB) for hypertension, loop diuretics for volume overload, and aldosterone receptor antagonists for reverse myocardial remodeling, as well as SGLT2 inhibitors (dapagliflozin/empagliflozin) and angiotensin-receptor neprilysin inhibitors to reduce the risk of HF hospitalizations or death in patients with preserved or mildly reduced LVEF [1]. Implantable cardioverter defibrillators (ICD) are indicated for patients with an LVEF < 35% at least 40 days after acute myocardial infarction (AMI). Another interventional option is cardiac resynchronization therapy (CRT) for patients with an LVEF < 35%, normal sinus rhythm, and QRS interval > 150 ms with a left bundle branch block (LBBB) [1,10,11,14,15]. 

In patients with acute decompensations, inotropes may reduce congestion, augment cardiac output, and aid peripheral perfusion. In patients who fail to respond to diuretic-based strategies, renal replacement therapy (RRT) should be considered with ultrafiltration as one of the most common approaches [1].

A minority of patients with advanced HF who are unresponsive to the above-mentioned therapy could potentially benefit from surgical therapy. Coronary artery bypass grafting (CABG) should be performed in patients with ischemic cardiomyopathy to reduce anginal symptoms, possibly improve ventricular function, alleviate symptoms of HF, and improve survival [10]. Ischemic mitral regurgitation (MR) is an important predictor of poor survival; thus, addressing it with a restrictive annuloplasty during CABG seems tempting. However, the long-term results of this concomitant approach remain controversial and inconclusive [15,16]. LV remodeling includes a number of procedures, all of which are based on Laplace’s law, i.e., achieving a reduction in wall tension via reducing LV radial dimensions and thus improving ventricular function. LV restoration is the most commonly used as it excludes dyskinetic and akinetic segments, reshaping the ventricle from its pathological spherical shape and restoring its more physiological elliptical shape [17]. 

When the patient has advanced HF and is not a candidate for any of the above-mentioned procedures or remains severely symptomatic despite them, more advanced mechanical circulatory support may be required. It is generally divided into short- and long-term MCS, with the former primarily reserved for INTERMACS profiles 1 to 3. Left ventricular assist device (LVAD) technology evolved from the 1st generation pulsatile chambers with volume displacements by external compression via the second generation axial continuous-flow spinning motors mounted on a central shaft to the modern third generation centrifugal continuous-flow hydrodynamic or electromagnetic suspended spinning motors, which are mostly used nowadays [5]. It is worth mentioning that in today’s practice, pulsatile devices are obsolete, and EM providers should not expect to feel any pulsations of arterial pressure when dealing with LVAD patients. LVADs are implanted in the majority of cases for two indications: as a bridge to transplantation or destination therapy, depending on the patient’s ability to fulfill strict criteria to become a heart transplant recipient (Table 4) [1]. These devices could be implanted on the cardiopulmonary bypass and via median sternotomy or even in a minimally invasive manner—via two separate mini-thoracotomies and without the artificial heart–lung apparatus [18,19]. They consist of an inflow cannula sutured to the LV, a spinning motor, a driveline, and an outflow cannula sutured (mostly) to the ascending aorta. The 13th annual report from the Society of Thoracic Surgeons (STS) INTERMACS highlights a 23.5% reduction in the annual volume in 2021 with 2464 primary LVAD implantations compared with peak implantation in 2019 and an ongoing trend from the prior year. This decline is likely a reflection of the untoward effects of the coronavirus disease 2019 pandemic. The last several years have been characterized by a shift in device indication and type, with 81.1% of patients now implanted as destination therapy and 92.7% receiving an LVAD with full magnetic levitation in 2021 [20].

## 5. Life-Threatening Complications of LVAD Therapy Potentially Leading to Cardiac Arrest

**Bleeding**. The most common adverse effect of continuous-flow MCS is gastrointestinal bleeding due to an abnormal von Willebrand factor degradation and de novo formation of arteriovenous malformations [3,21]. Other factors, such as the utilization of anticoagulants and antiplatelet agents, the degree of the inflammatory response, the presence of angiodysplasia, and possibly even blood group type may also play an important role in the pathophysiology of post-implantation bleeding [4]. Regardless of the source of bleeding, cardiac tamponade or significant hemothorax can result in hemodynamic instability from either obstructive or hypovolemic shock, prompting urgent thoracentesis or surgical re-exploration. Life-threatening bleeding in LVAD patients requires early anticoagulation reversal, ideally with vitamin K or four-factor prothrombin complex concentrate, with the latter preferred for warfarin reversal due to smaller volume, faster administration time, and faster restoration of coagulant factor activity in comparison to fresh frozen plasma and fewer pump thromboses and undemanding coagulation status monitoring in comparison to vitamin K [22]. Due to the high association of bleeding complications with preceding infections, empiric antibiotics should be strongly considered in patients with bleeding complications [23]. 

**Infection**. Infectious complications are frequently encountered as they represent the second highest event rate and can involve any portion of LVAD—surgical site, driveline (being the most prevalent), pocket, or the pump itself [5]. Since these infections tend to persist due to biofilm formation, antibiotic penetration is limited and insufficient if given alone—either negative pressure wound therapy or surgical replacement is often warranted. Regardless of the origin of the infection, LVAD patients can develop septic (distributive) shock requiring intensive fluid supplementation and/or vasoactive drugs [24]. Three sets of blood cultures and a sterile percutaneous wound culture should be obtained prior to initiating antibiotics, which should cover methicillin-resistant S. aureus, pseudomonas, coagulase-negative staphylococci, and fungi [25]. 

**Stroke**. Not uncommonly observed, a significant neurological event may result in a patient being found unresponsive despite a normally functioning device. The highest risk for any kind of cerebrovascular insult is high fluctuation of antithrombotic therapy ranging from insufficient to excessive [4,26]. EM healthcare providers should pay extra attention to an unconscious patient without a pulse and with the slightest hint of any LVAD implantation [24,26]. Endovascular therapies are prioritized, as thrombolytics are generally contraindicated [27]. 

**Pump failure**. The two most common causes of pump failure are disconnection of the power and failure of the driveline. Therefore, in assessing an unresponsive, mentally altered, or hypotensive patient with an LVAD, it is critically important to ensure that all connections are secure, and an adequate power source is connected. Patients with a current LVAD system typically have two batteries connected to power the device—both should be checked for their status. Controller malfunction, damage, or disconnection can also lead to pump dysfunction/stoppage. Additionally, the driveline is a potentially vulnerable component and subject to wear, damage, or kinking. In these settings, there will often be alarms preceding or accompanying the pump stoppage, but alarms will cease once the batteries are drained [24]. It is important to establish the correct cause of the malfunction, which, in most cases, requires device exchange. Another devastating adverse effect potentially leading to pump failure is pump thrombosis, which is associated with high morbidity and mortality [3,21,26]. The first sequela of pump thrombosis is usually clinically significant hemolysis (significant lactate dehydrogenase (LDH) elevation); however, it may also result in stroke, peripheral embolism, worsening HF, or death [4]. In the case of pump thrombosis, hydration, urine alkalization, and early anticoagulation with unfractionated heparin or bivalirudin should be initiated. Unfortunately, this combination of treatments alone has been shown to fail in up to 40%, thus exhibiting the need for surgical pump exchange [28]. Systemic thrombolysis may be a reasonable option for life-threatening pump thrombosis in the destination therapy group, where device exchange is often impossible due to their existing comorbidities [29]. 

## 6. Approach to Cardiopulmonary Resuscitation in Patients with LVAD

**Figure 1:**
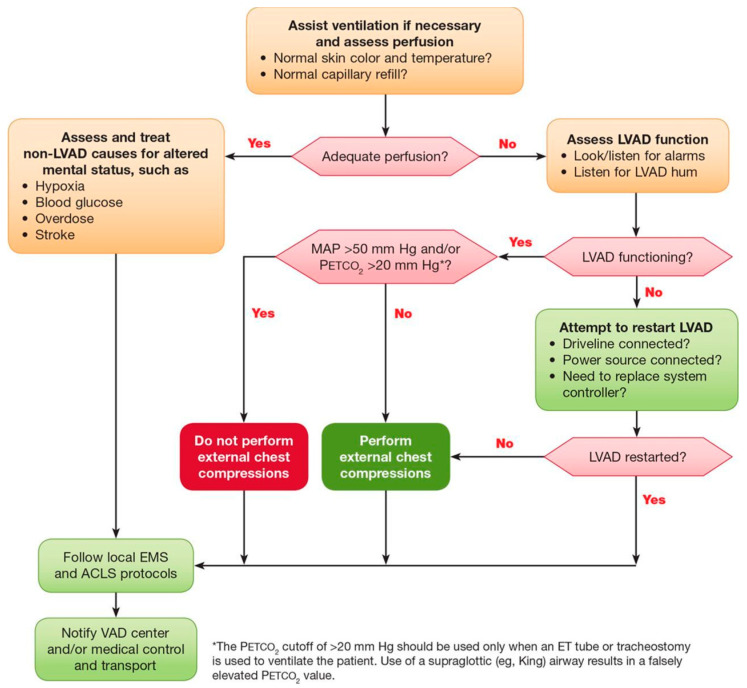
Algorithm showing response to a patient with a left ventricular assist device (LVAD) with unresponsiveness or other altered mental status (reprinted with permission). Note: LVAD: left ventricular assist device; ACLS: advanced cardiac life support; EMS: emergency medical service; PETCO_2_: partial pressure end-tidal carbon dioxide; ET: endotracheal; MAP: mean arterial pressure.

Regarding out-of-hospital cardiac arrest, the American Heart Association (AHA) issued its scientific statement regarding CPR in adult and pediatric MCS population [24]. Their algorithm of medical response to an unresponsive or otherwise mentally altered patient on LVAD is shown in Figure 1. 

### 6.1. Assessment of Flow and Perfusion

After assisting with ventilation, if necessary, the next step should always be an assessment of flow and perfusion in mentally altered patients with LVAD. This could be achieved clinically via skin color and capillary refill inspection, followed by non-invasive blood pressure measurement using a manual blood pressure (BP) cuff and a Doppler. A manual BP cuff alone is unreliable more than 50% of the time. When using a manual Doppler, pressure measurement is accomplished by attaching a manual BP cuff to the patient’s arm, inflating to at least 120 mm Hg systolic, and slowly deflating while placing a Doppler probe over the radial artery [30,31]. Early proceeding to the arterial line for invasive BP monitoring should follow. Another pliable option represents waveform capnography, otherwise routinely used during cardiopulmonary resuscitation by EM healthcare providers [32]. A decrease in end-tidal carbon dioxide (etCO_2_) in intubated patients correlates with systemic hypoperfusion, low cardiac output, and low venous return [24,33]. After the return of spontaneous circulation (ROSC), an increase in etCO_2_ is noticed. Pulse oximetry can be unreliable due to the lack of pulsatility; obtain an arterial blood gas early if the peripheral capillary oxygen saturation has a poor waveform [34]. 

### 6.2. Assessment of Device

The next step after assessing perfusion is assessing the device itself. If LVAD is determined as a destination therapy with a clear do-not-resuscitate notion, the same protocol as for other non-LVAD patients should be applied. If it is placed as a bridge to transplantation/decision/candidacy/recovery, active management is undertaken. The device should be assessed for function by looking and listening for alarms and listening for an LVAD hum over the left chest and left upper abdominal quadrant. The two most often reversible causes of pump failure are a failed connection linking the controller with the pump and inadequate electrical power. Proceed with ensuring secure connections to the controller and ensuring adequate power for the LVAD via an additional power source or external energy source (wall outlet with adaptor). LVAD patients usually carry a spare controller and additional power source. Failure to identify a repairable cause of device malfunction usually results in unsuccessful outcomes [35]. 

### 6.3. Chest Compressions and Defibrillation

When LVAD functions correctly and with a low etCO_2_ as well as low arterial pressure, chest compressions are indicated (Figure 1). CPR is encouraged in LVAD patients, but instead of using a pulse vs. pulseless algorithm, initiate resuscitation based on perfusion status, device failure, arterial pressure, or etCO_2_ [36]. An active and aggressive search for reversible causes of cardiac arrest (4H/4T) should be conducted. Cardioversion and defibrillation pads should not be placed directly over the pump; however, in case of hemodynamically significant arrhythmias, no delay in cardioversion or defibrillation should occur as patients are often refractory to medical therapies [37]. 

### 6.4. Pump Alarms

As per Figure 1, in case of adequate perfusion, meaning MAP above 50 mmHg, chest compressions are not indicated. Pump alarms should be evaluated. LVAD flow alarm can signal either low or high flow deviation.

When low-flow alarm, causes of hypovolemia (dehydration, bleeding, and right-sided HF) should be considered. Paradoxically, hypertension could also cause low flow as afterload increases and LVADs are afterload-dependent. 

The high-flow alarm could be combined with normal power, thus directing our investigation toward possible sepsis or other distributive shock causes, or with high power, potentially indicating pump thrombosis [38]. 

A word of caution: the “cardiac output” number on the LVAD screen is a calculation based on the speed and power consumption of the unit and represents true flow only in a state of proper functioning. Any cause of hypotension (bleeding, hemolysis, or anemia) will result in elevated screen numbers to alleviate the drop in systemic vascular resistance. Thus, the appropriate clinical examination should lead the EM provider’s management [24]. 

### 6.5. Additional Diagnostic Assessment

Another cornerstone in the evaluation of an arresting LVAD patient is transthoracic echocardiography, performed either by an experienced EM provider or a consultant cardiologist. Most important dilemmas should address RV filling (preload) and function, valve competence, degree of LV unloading, and if there are any obstructing thrombi within the pericardial cavity or the device itself [39,40]. 

Obtaining an electrocardiography (ECG) strip provides us with additional knowledge about the electrical activity of the heart. Ventricular arrhythmias occur in 22–59% of LVAD patients. Although it is not uncommon for patients to be asymptomatic during ventricular tachycardia or ventricular fibrillation, it is critical to terminate those arrhythmias since the risk of right ventricular failure is high. Interpretation of ECG could be difficult due to electromagnetic interference of the pump that causes high-frequency noise artifacts on 12-lead ECGs. Elimination of noise can be achieved by adjusting filtering parameters [41]. 

Chest radiographs should be obtained since they provide information on appropriate device placement. A more accurate computer tomography imaging can provide us, in addition to LVAD position itself, data on other pathological events such as the origin of the infection, mediastinal hematomas, pericardial, abdominal wall, and retroperitoneal hemorrhage, inflow and outflow graft and aortic thrombi [42].

Importantly, patients with LVAD should not undergo magnetic resonance imaging due to the highly ferromagnetic component incompatibility [43]. 

B-type natriuretic peptide (BNP) levels decrease after LVAD implantation due to LV unloading. Acute increases in BNP from the new (post-implantation) baseline may occur during situations of inadequate circulatory support, such as pump failure or tamponade [44]. 

### 6.6. Transfer to Implanting Facility

It should be noted that all patients receive the LVAD coordinator’s contact number at the time of LVAD implantation, and this should remain the early referral connection when being treated by an EM provider. After the above-described primary assessment, simultaneously, local EMS and ACLS protocols are followed while contacting the LVAD coordinator and transferring to the implanting facility. After achieving ROSC, all LVAD patients should be treated in the implanting facility for a higher level of care and definite management. 

## 7. Long-Term Results

The long-term results of this patient cohort are scarcely reported in the scientific literature. The largest report looked at a recent period of 8 years in the US and identified 93,153 hospitalizations with implanted LVAD. Only 578 patients had experienced cardiac arrest, and 173 patients (33%) among these underwent CPR. In-hospital mortality was 60.8% in patients with cardiac arrest (including do-not-resuscitate LVAD patients) and, interestingly, 74.33% in patients in whom CPR was performed. This resulted in a trend toward fewer CPR performances toward the end of this period. Additionally, age, female sex, peripheral vascular disease, history of CABG, and ventricular tachycardia were independently associated with in-hospital mortality [45]. Regarding life-threatening LVAD complications, the MOMENTUM 3 trial showed fewer strokes, major bleedings, and reoperations to replace or remove a malfunctioning device as well as lower mortality for third generation centrifugal continuous-flow pumps than second generation axial continuous-flow devices [46]. Recently, data from a retrospective cohort study in n = 337 patients who received an LVAD after extra-corporeal life support showed that the 30-day, 1-year, and 3-year survival were 87%, 73%, and 65%, respectively [47].

## 8. Future Challenges

Certain unclarities persist, such as (I) what are the risks/benefits of chest compressions to patients with an LVAD?; (II) does etCO_2_ alone act as a good identifier of patients with (sub)optimal cardiac output; and (III) what kind of identification should patients with LVADs wear on themselves to avoid any uncertainties regarding the presence of an MCS device? Taking into account that no randomized clinical trial could be feasible and ethically justified in this patient population, HF guidelines for the management of out-of-hospital cardiac arrest in patients with an LV assist device will most probably lean on “expert consensus” rather than “evidence-based” data also in the foreseeable future. 

## 9. Conclusions

When approaching a patient on LVAD with a suspected cardiac arrest, obtain a manual Doppler pulse measurement and plug the device into an external battery source. Auscultation over the chest to listen for a machinery whirring sound should be quickly performed, and CPR should be initiated when clinical signs of hypoperfusion along with decreased arterial pressure are noted. Cardioversion and defibrillation are compatible with LVADs as long as the pads are not placed directly over the pump. The endpoint of resuscitation is clinical signs of improvement with sufficient etCO_2_ and arterial pressure. After ROSC, initiation of standard post-resuscitation circulation care should be carried out with inotropic support with catecholamines if indicated. All LVAD patients should be transferred to the implanting facility for definite management. 

## Figures and Tables

**Table 1:** Criteria for definition of advanced heart failure (modified from [7]).

1.	Severe and persistent symptoms of HF;
2.	Severe cardiac dysfunction: LVEF < 30% or inoperable valve abnormalities or inoperable congenital malformations or severe LV diastolic dysfunction;
3.	Episodes of pulmonary/systemic congestion or low output requiring vasoactive support or malignant arrhythmias;
4.	Severe impairment of exercise capacity.

Note: HF, heart failure; LVEF, left ventricular ejection fraction; LV, left ventricular.

**Table 2:** New York Heart Association functional classification (modified from [12]).

Class 1	No limitation of ordinary physical activity.
Class 2	Slight limitation of ordinary physical activity.
Class 3	Marked limitation of ordinary physical activity, but comfortable at rest.
Class 4	Unable to carry out physical activity, symptomatic at rest.

**Table 3:** INTERMACS level of limitation at time of implant (modified from [13]).

INTERMACS Profile	Description	Time Frame for Intervention
1	Critical cardiogenic shock (life-threatening hypotension, critical organ hypoperfusion). “*Crash and burn*”.	Within hours
2	Progressive decline (worsening renal function, nutritional depletion). “*Sliding on inotropes*”.	Within days
3	Stable but inotrope-dependent (repeated failure to wean from inotrope support due to recurrent hypotension or renal failure). “*Dependent stability*”.	Within weeks
4	Resting symptoms (daily symptoms of congestion at rest or during activities). “*Frequent flyer*”.	Within months
5	Exertion intolerant (comfortable at rest, unable to engage in any other activity). “*Housebound*”.	Variable urgency, depends on maintenance of nutrition, organ function, and activity.
6	Exertion limited (comfortable at rest and with activities of daily living, but fatigue shortly after commencing any meaningful activity). “*Walking wounded*”.	Variable urgency, depends on maintenance of nutrition, organ function, and activity.
7	Intermediate NYHA III to IV.	MCS may not currently be indicated.

Note: INTERMACS, Interagency Registry for Mechanically Assisted Circulatory Support; NYHA, New York Heart Association; MCS, mechanical circulatory support.

**Table 4:** Terms describing various indications for mechanical circulatory support (modified from [1]).

Bridge to decision	Short-term MCS until hemodynamics and end-organ perfusion are stabilized.
Bridge to candidacy	Long-term MCS to improve end-organ function to make an ineligible patient eligible for a heart transplant.
Bridge to transplantation	Long-term MCS to keep a patient without contraindications for heart transplant alive until a donor’s heart is available.
Bridge to recovery	Short- or long-term MCS to keep a patient alive until cardiac function recovers sufficiently.
Destination therapy	Long-term MCS as an alternative to heart transplant in patients with contraindications for transplant.

MCS: mechanical circulatory support.

## Data Availability

The data used to support the findings of this study are available from the corresponding author upon request.
